# Spontaneous Evisceration of Incisional Hernia with Strangulation of Small Bowel: A Life Threatening Complication

**DOI:** 10.1155/2021/6684360

**Published:** 2021-02-02

**Authors:** Nanduni Thalahitiyage, Sanjeevan Ravindrakumar, Janaka Nandasena, Arooran Krishnakumar, KirushanthanVeerasingam, Umesh Jayarajah, V. S. D. Rodrigo

**Affiliations:** Department of Surgery, District General Hospital Chilaw, Chilaw, Putlam District, Sri Lanka

## Abstract

Spontaneous evisceration of abdominal viscera is a rare complication of incisional hernia which could pose a serious threat to life if intervention is delayed. We report a case of a 62-year-old female with a history of curative resection for stage 1 endometrial adenocarcinoma 3 years ago, presenting with spontaneous evisceration of incisional hernia with strangulation of small bowel. Immediate resuscitation followed by emergency surgery was mandatory. During surgery, priority should be given to release the strangulation as soon as possible and the type of repair would depend on the viability of the bowel and the anatomy of the incisional hernia.

## 1. Introduction

Incisional hernias arise through a defect in the musculofascial layers of the abdominal wall in the region of a postsurgical scar. Spontaneous evisceration of abdominal viscera is a rare complication of incisional hernia which could pose a serious threat to life if intervention is delayed [1–3]. We report a case of a spontaneous evisceration of incisional hernia with strangulation of small bowel.

## 2. Case Presentation

A 62-year-old obese female presented with spontaneous rupture of incisional hernia for 4 hours duration. She had a history of curative total abdominal hysterectomy and bilateral oophorectomy for stage 1 endometrial adenocarcinoma 3 years ago. She was also treated with adjuvant chemoradiation. Subsequently, she developed a surgical site infection and with breakdown of skin and subcutaneous tissues and was treated with repeated wound debridement and secondary suturing. She did not have diabetes mellitus or other long-standing medical comorbidities. She had noticed a bulge in the lower abdomen which was progressively enlarging in size over a period of 7 months and did not seek treatment.

**Figure 1:**
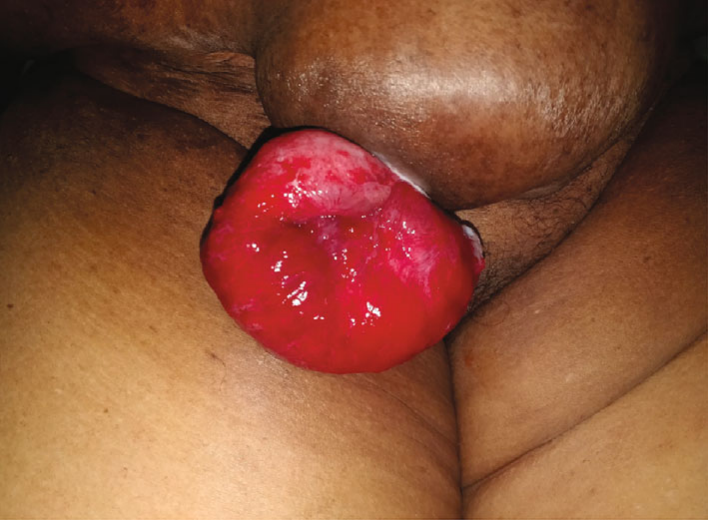
Preoperative image showing evisceration of small bowel with evidence of strangulation.

On admission, she did not have fever or features of intestinal obstruction. She was haemodynamically stable. Her abdomen was pendulous with a large incisional hernia over the Pfannenstiel incision. A loop of grossly congested and oedematous small bowel was seen eviscerating through a small defect in the skin suggestive of early strangulation (Figure 1).

Her blood investigations including full blood count, serum electrolytes, renal functions, and clotting profile were normal. She was resuscitated and planned for emergency surgery.

**Figure 2:**
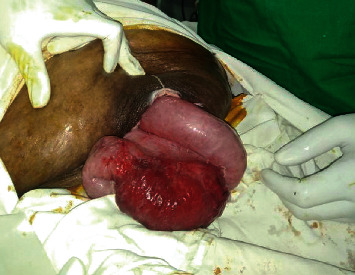
Intraoperative image showing viable small bowel after releasing the strangulation.

As the first step during surgery, the strangulation was released by enlarging the skin defect which was 3 cm in diameter (Figure 2). Fortunately, the bowel was viable without any evidence of gangrene. Thereafter, an elliptical incision was made over the incisional hernia incorporating the defect. A large (10 cm in diameter) abdominal wall defect was identified with extensive adhesions. Careful adhesiolysis was performed and the bowel was reduced into the peritoneal cavity. The defect was closed and an on lay mesh repair was performed. She had an uneventful recovery and was discharged on the 5^th^ postoperative day. At one month following surgery, she had complete wound healing and was able to perform her normal day-to-day activities.

## 3. Discussion

We report an unusual presentation of spontaneous rupture and evisceration of incisional hernia with strangulation of small bowel. This is a rare complication of an abdominal incisional hernia, which is a defect in the abdominal wall that lies close to a scar from a previous full-thickness abdominal incision [2, 3]. Similar case reports published after 2010 are summarized in Table 1. Similarly, rupture and evisceration of parastomal hernias have also been reported [4]. There are multiple risk factors for developing incisional hernias, such as advanced age, wound infection, diabetes mellitus, poor nutrition, malignancy, obesity, and any condition that causes a chronically raised abdominal pressure [5]. Our patient had several risk factors such as older age, obesity, malignancy and history of wound infection, and repeated wound debridement following initial surgery. Furthermore, the adjuvant chemoradiation would have also resulted in poor wound healing and caused the development of the incisional hernia.

In the majority, incisional hernias are identified incidentally on examination. Nevertheless, delay in repair of incisional hernias can lead to major complications such as bowel incarceration, strangulation, ischaemia, and even bowel necrosis and perforation [2]. However, spontaneous rupture and strangulation is an unusual complication [5].

Spontaneous rupture of an incisional hernia may be sudden or gradual. Sudden rupture occurs due to a steep rise in pressure in the abdominal cavity (e.g., coughing and lifting heavy objects) [6]. On the contrary, gradual rupture occurs following an ulcer on the dependent part of the sac of the hernia. Large incisional hernias are more prone to rupture due to the thin hernia sac, with atrophied overlying skin [6].

Eviscerated hernias must be treated immediately in order to prevent bowel strangulation and obstruction. If the bowel is viable, primary mesh repair may be performed. As an alternative, primary closure without a mesh followed by delayed secondary mesh repair may also be performed [7]. In the event of bowel gangrene, a laparotomy with bowel resection and anastomosis would be required [7]. We performed a primary mesh repair, because the bowel was viable after releasing the strangulation and the patient had a satisfactory outcome. An onlay mesh repair was performed as opposed to a retrorectus repair as we were more familiar with the onlay technique, and furthermore, the rectus sheath was considerably thinned out making retrorectus dissection difficult.

## 4. Conclusion

We report a patient with an incisional hernia who presented with spontaneous evisceration and strangulation of small bowel. Immediate resuscitation followed by emergency surgery was mandatory. During surgery, priority should be given to release the strangulation as soon as possible, and the type of repair would depend on the viability of the bowel and the anatomy of the incisional hernia.

## Figures and Tables

**Table 1:** Summary of previous similar case reports published after 2010 [1, 2, 8–17].

Author (year) country	Gender	Age	Clinical features	Investigations	Management	Follow-up
Das [8] (2011) India	Female	53	History of laparoscopic cholecystectomy five years ago. Developed acute severe abdominal pain over an umbilical hernia with nausea and anorexia. Abdominal examination-abdominal distension with large umbilical hernia with ulceration and intestinal evisceration.	None	Resuscitation and nasogastric decompression. Emergency exploration-Infraumbilical smiling incision (inflamed appendix, part of caecum, terminal ileum found as contents). Intestine appeared viable and returned to the abdominal cavity. Appendectomy done. Adhesions released and fascia sutured with polypropylene suture	Drain removed on 2nd postoperative day and discharged on 6th postoperative day
Akkucuk [9] (2013) Turkey	Female	65	History of C-section 30 years back and four incisional hernia repairs: last repair done 20 months back. Large incisional hernia in the right groin and a short segment of intestine eviscerated through the hernia.	Ultrasonography: intestinal segments protruded to the right side of groin. CT scan of lower abdomen-herniation of intestinal segments and mesenteric lipomatous tissue to the subcutaneous area through the muscular defect in the right lower abdominal quadrant.	Segmental resection and end-to-end ileoileal anastomosis was performed. Hernia gap was repaired with primary sutures along with a prosthetic mesh repair. Subcutaneous suction drain kept and wound closed.	Drain removed on 2nd postoperative day and discharged after 5 days. Reexamination in 6 months revealed no signs of hernia recurrence
Umadevi [1] (2013) India	Female	50	History of hysterectomy and pelvic lymph node dissection with midline abdominal incision for carcinoma endometrium 1.5 years back (defaulted for adjuvant therapy and follow-up). Presented with burst abdomen and evisceration of bowel following bending.	None	Emergency laparotomy: findings revealed 8 cm rectus sheath defect with gangrenous bowel loop protruding from the skin gangrenous small bowel was resected and end to end anastomosis was done. Anatomical repair of the hernia was done.	3 cm marginal flap necrosis noted postoperatively which was debided and secondary suturing was done. Patient was asymptomatic until 2 months of follow-up.
Agodirin [10] (2015) Nigeria	Female	34	History of two previous caesarean sections: last being done 10 months back. Dehiscence of the wound following the surgery and appearance of an incisional hernia six months later. At presentation-bowel loops protruding through the distal part of a lower midline hypertrophic scar.	None	Emergency exploration-eviscerated bowel loops irrigated and returned to the abdominal cavity. Defect closed with nonabsorbable interrupted stitches	Followed up for over 12 months without evidence of recurrence
Roy [11] (2015) India	Female	55	History of total abdominal hysterectomy done 6 years prior ruptured incisional hernia with evisceration of small gut through the lower half of infraumbilical vertical scar	None	Surgical exploration: hernial contents reduced gap in the rectus sheath repaired anatomically with polypropylene sutures	Uneventful recovery discharged on the 11th postoperative day no complication or recurrence noted after one year of follow-up
Osei-Tutu [2] (2016) Ghana	Female	56	History of abdominal surgery over 20 years ago. Presented with acute abdominal pain, multiple episodes of vomiting and protrusion of 40 cm of small bowel through an incisional hernia for 4 hours. Chronic cough associated with weight loss for 6 months was noted.	Haemoglobin: 11.1 g/gl WBC: 8 × 10^9^/L Platelets: 238 × 10^3^/L	Resuscitation and emergency surgery. Laparotomy-Adhesiolysis and reduction of the bowel to the peritoneal cavity and fascia closed with nylon.	Recovery was uneventful. Diagnosed to be positive for HIV 1 and referred to the medical team. Discharged on the 4th postoperative day and lost to follow-up.
West [12] (2016) UK	Male	70	History of emergency abdominal aortic aneurysm repair with a development of a large incisional hernia. Presented with spontaneous rupture of incisional hernia with his appendix and greater omentum protruding out.	CT scan: large defect measuring 18 × 15 cm containing the bowel, omentum, and evisceration of the appendix	Emergency incisional hernia repair with open appendectomy. Collagen mesh was used to achieve closure of the myofascial defect. Abdominal binder was applied.	Discharged on 6th postoperative day. Review after 7 weeks showed no evidence of recurrence or infection.
Ansari [13] (2017) India	Female	40	History of emergency laparotomy for tubercular ileal perforation 10 years ago. Bowel protrusion from a ruptured ventral incisional hernia. Hypertensive (206/158 mmHg), tachycardic (106 beats per minute), and pale on presentation.	Full blood count, renal functions and blood sugar were normal. Arterial blood gas: mild respiratory alkalosis.	Nitroglycerine intravenous infusion to lower the blood pressure. Emergency surgery: a loop of small bowel was found adherent to superior aspect of the hernial defect and was dissected free. Hernial sac with redundant skin was excised and large hernial defect of about 10 × 8 cm in size was closed by polypropylene mesh underlay repair	Postoperative period was uneventful except for a minor stitch abscess at the lower end of the wound that cleared in 3 days. Discharged on the 8th day at 2-year follow-up, no recurrence was noted.
Edeh [14] (2018) Nigeria	Female	56	History of laparotomy and abdominal hysterectomy 1 year back and appearance of hernia after 3 months of surgery. Had acute pain and evisceration of the bowel for 30 minutes. Abdominal examination-large swelling of >20 cm in diameter in the upper half of subumbilical midline scar and evisceration of two loops of jejunum.	Haemoglobin-10.3 g/dL; WBC: 5 × 10^9^/L; Platelets: 340 × 10^9^/L	Resuscitation and emergency surgery. Surgery: hernial sac was dissected and returned to the peritoneal cavity with the bowel. The defect was enlarged by a midline incision up to the umbilicus and down to the pubis and closed with nylon. A 15 × 15 cm polypropylene mesh was sutured to the closed defect.	Postoperative period was uneventful and discharged on the 5th postoperative day. Follow-up at 6 months showed no signs of recurrence
Thakkar [15] (2019) India	Female	35	History of laparotomy 8 years back and development of an incisional hernia 2 years prior. Presented with omentum protruding through the anterior abdominal wall when she was lifting a weight.	None	Surgical exploration: central part of thinned out sac and sheath was found. After excising the omentum, abdominal viscera were examined and were normal. Primary closure of the sheath was done. Plane created for meshplasty and done with a prolene mesh.	Postoperative period was uneventful. Wound healed well without any infection.
Lim [16] (2020) Singapore	Male	65	Past history of primary surgical repair of a strangulated umbilical hernia 4 years prior with subsequent exploratory laparotomy for postoperative adhesions. Significant past medical history of Child–Pugh's B liver cirrhosis, with complications including portal hypertension, esophageal varices, and ascites. Bed bound patient. Hypothermic, hypotensive, tachypneic on presentation with irreducible eviscerated congested small bowel from a skin defect of 4 cm, with impingement of the mesentery.	None	Resuscitation with emergency surgery, skin defect extended as a limited midline laparotomy incision. 2.5 L of ascitic fluid drained. Congested but viable small bowel loops were reduced into the peritoneal cavity. Biologic mesh was fashioned in 15 cm × 20 cm size and anchored in-lay to the widely retracted fascia	Patient did not experience any hernia recurrence. Passed away after 2 years from pneumonia.
Weledji [17] (2020) West Africa	Female	55	Past history of hysterectomy 12 years prior complicated by urinary fistula for which laparotomy and repair was done. Development of incisional hernia few years later managed with an abdominal corset. Presented with one-week history of progressive abdominal pain, vomiting, and absolute constipation. On examination: proximal small bowel obstruction secondary to a tender, large, and irreducible incisional hernia with a faecal fistula at its apex	Full blood count and serum biochemistry normal were normal.	Resuscitation with fluids and nasogastric decompression. Laparotomy grossly dilated small bowel loops incarcerated in an incisional hernial sac. Hernial sac was excised en bloc with the incarcerated and fistulating ileal loop. Ileoileal anastomosis was made and hernia defect was repaired with Jenkin's mass closure suture technique.	Recovery complicated by a copious seroma for a week that led to superficial wound dehiscence and mild wound infection—managed with daily dressings and 1-week course of broad-spectrum intravenous antibiotics. Discharged two weeks after surgery. At three months, wound was completely healed and no evidence of recurrence was noted.

## Data Availability

The data used to support the findings of this study are included within the article.
